# Dapsone reduced cuprizone-induced demyelination via targeting Nrf2 and IKB in C57BL/6 mice

**DOI:** 10.22038/IJBMS.2022.64993.14310

**Published:** 2022-06

**Authors:** Ahmad Reza Dehpour, Ehsan Khaledi, Tayebeh Noori, Ahmad Mohammadi-Farani, Ladan Delphi, Antoni Sureda, Eduardo Sobarzo-Sanchez, Samira Shirooie

**Affiliations:** 1 Department of Pharmacology, School of Medicine, Tehran University of Medical Sciences, Tehran, Iran; 2 Experimental Medicine Research Center, Tehran University of medical sciences, Tehran, Iran; 3 Student Research Committee, Faculty of Pharmacy, Kermanshah University of Medical Sciences, Kermanshah, Iran; 4 Pharmaceutical Sciences Research Center, Health Institute, Kermanshah University of Medical Sciences, Kermanshah, Iran; 5 Medical Plant Research Center, Basic Health Sciences Institute, Shahrekord University of Medical Sciences, Shahrekord, Iran; 6 Department of Physiology and Pharmacology, School of Medicine, Shahrekord University of Medical Sciences, Shahrekord, Iran; 7 Animal Biology Department, Faculty of Biology, College of Sciences, University of Tehran, Tehran, Iran; 8 Research Group on Community Nutrition and Oxidative Stress (NUCOX) and Health Research Institute of Balearic Islands (IdISBa), University of Balearic Islands, Palma de Mallorca E-07122, Balearic Islands, Spain; 9 CIBER Fisiopatología de la Obesidad y Nutrición (CIBEROBN), Instituto de Salud Carlos III (ISCIII), 28029 Madrid; 10 Instituto de Investigación y Postgrado, Facultad de Ciencias de la Salud, Universidad Central de Chile, Chile; 11 Department of Organic Chemistry, Faculty of Pharmacy, University of Santiago de Compostela, Spain

**Keywords:** Cuprizone, Dapsone, Multiple sclerosis, Neuroinflammation, NF-kB, Nrf2

## Abstract

**Objective(s):**

Multiple Sclerosis (MS) is an inflammatory disorder wherein the myelin of nerve cells in the central nervous system is damaged. In the current study, we assessed the effect of Dapsone (DAP) on the improvement of behavioral dysfunction and preservation of myelin in the cuprizone (CPZ) induced demyelination model via targeting Nrf2 and IKB.

**Materials and Methods:**

MS was induced in C57BL/6 mice through diet supplementation of CPZ (0.2%) for 6 weeks, and DAP (12.5 mg/kg/day; IP) was administered for the last 2 weeks of treatment. Pole test and rotarod performance test, LFB and H&E staining, and Immunohistochemistry (IHC) staining of p-Nrf2 and p-IKB were performed. Furthermore, superoxide dismutase (SOD) and nitrite were measured.

**Results:**

DAP treatment prevented body loss induced by CPZ (*P<*0.001). Pole test showed that CPZ increased latency time to fall (*P<*0.0001) but the latency to reach the floor in the DAP-CPZ group was significantly shorter (*P<*0.0001). Rotarod performance test showed the effect of CPZ in reducing fall time in the CPZ group (*P<*0.0014); however, DAP significantly increased fall time (P=0.0012). In LFB staining, DAP reduced demyelination induced by CPZ. CPZ significantly decreased p-Nrf2 and elevated p-IKB levels compared with the control group (*P<*0.0001), but in DAP-treated groups markedly modified these changes (*P<*0.0001). CPZ increased the brain nitrite levels and reduced SOD activity, but in DAP-treated considerably reversed CPZ-induced changes.

**Conclusion:**

These data support the suggestion that the beneficial properties of DAP on the CPZ-induced demyelination are mediated by targeting Nrf2 and NF-kB pathways.

## Introduction

Multiple sclerosis (MS) is a long-lasting autoimmune demyelinating disease of the brain and spinal cord that produces a variety of symptoms such as fatigue, incoordination, blurred vision, and psychiatric problems (1). Current immune-modulating drugs such as interferon beta (IFN-β) and natalizumab used in the treatment of MS have low efficacy and high side effects. The serum and tissue level of nitrite as a non-specific inflammatory mediator of oligodendrocyte loss have been associated with MS (2, 3). Generally, the aim of the treatment of MS with these drugs is to slow the progress of the disease and decrease relapse (4). 

Biscyclohexanone oxaldihydrazone (cuprizone) (CPZ), a copper (Cu) chelator, has neurotoxic effects which can damage the white matter of the CNS (5). In 1966, Carlton detected that CPZ administration induced demyelination in mice (6). Several studies have shown that CPZ-treated mice show extensive demyelination, metabolic stress, and oligodendrocyte apoptosis (7, 8). The mouse CPZ-induced model is usually utilized to study remyelination and demyelination because of its unique pathological mechanisms, and CPZ is vital to simulate the key activities of myelin-forming oligodendrocytes (OLs) in remyelination and demyelination (9). Mice fed with 0.2% CPZ between 4 to 6 weeks revealed demyelination and had abnormal behavior like fewer social interactions (10, 11). The pathological effects of CPZ are based on many reasons such as (a) disruption of Cu homeostasis (5), (b) inhibition of enzymes (12), (c) megamitochondria due to fission inhibition, and generation of free radicals, (d) increased oxidative stress due to declined function of the antioxidant enzymes catalase and superoxide dismutase (SOD), reduction of glutathione (GSH) levels, elevation of Fe^+2^ in mitochondrial matrix and cytosol and lipid peroxidation (5, 13), and (e) increases of the activity of plasmalogens and phospholipase A2 (PLA2) and arachidonic acid (AA) which can produce prostaglandins and leukotrienes (14). One of the main pathological effects of CPZ is a significant reduction of expression and activity of Nrf_2_, a cytoprotective factor, that leads to oligodendrocyte loss (15). So, CPZ-induced demyelination is used as a toxic experimental model of demyelination of neurons with similar pathology to human MS (16).

Nuclear factor erythroid 2-related factor 2 (Nrf_2_) is a transcriptional factor that controls the expression of anti-oxidant proteins and protects the cells against oxidative stress during inflammation and injury (17). During normal conditions, Nrf_2_ present in the cytosol is inactivated by binding to kelch-like ECH-associated protein 1 (Keap1). During oxidative stress injury, Keap1 is oxidized and dissociated from the Keap1-Nrf_2_ complex, allowing Nrf2 to be activated by phosphorylation, becoming the biologically active form. Once activated, p-Nrf_2_ goes to the nucleus and increases the expression of antioxidant factors by binding to consensus antioxidant response elements (AREs) (18). Some of the Nrf_2_-regulated antioxidants are quinine oxidoreductase-1 (NQO-1) and heme oxygenase-1 (HO-1) (19). Some reports indicate that in demyelinating abrasions of MS subjects, the activity of Nrf_2_, as well as the levels of NQO-1 and HO-1, were reduced (15, 20). Thus, up-regulation of Nrf_2_ may be helpful to MS therapy due to induction of protective enzymes and reduction of oxidative stress.

Dapsone (DAP), a diaminodiphenyl sulfone, exerts antimicrobial and anti-protozoa effects (21). This sulfonamide-related drug is one of the vital medications for the treatment of leprosy (22). Besides, DAP is used to treat *Pneumocystis carinii* pneumonia, malaria, acne, and many dermatologic disorders (23, 24). It has been reported that DAP has anti-inflammation, antioxidant and neuroprotective properties (25-28). An *in vivo* study on rat colitis induced by acetic acid has indicated that DAP ameliorates oxidative stress, reducing the levels of malondialdehyde (MDA), and increasing the activity of SOD and it can also decrease pro-inflammatory cytokines as well as the level of p-NF-kB (29). 

Based on these previous data, the purpose of this research was to evaluate the therapeutic effect of DAP in reducing the CPZ-induced demyelination in C57BL/6 mice by rotarod and pole tests. Some oxidative stress factors such as SOD and nitrite and the levels of p-IKB and p-Nrf_2_ were also determined in the brains of animals. 

## Materials and Methods

***Animals***

Twenty-one adult male C57BL/6 mice (20-25 g) were attained from the Pasteur Institute (Karaj, Iran). Mice were kept at 22±1 ^°^C, 12 hr/12 hr light/dark cycle with free admittance to food and water. All trials were accepted by the Ethics Committee of Tehran University of Medical Science (TUMS), Tehran, Iran.

***Induction of cuprizone-demyelination and dapsone treatment***

To induce demyelination, mice were fed 0.2% (w/w) CPZ (Merck, Germany) in their chow for six weeks (5, 30). Animals were randomly divided into 3 groups (7 mice in each) as follows:

1. Control group: fed with standard diet for six weeks.

2. CPZ group: fed with a diet supplemented with 0.2% (w/w) CPZ for six weeks.

3. DAP-treated CPZ group: fed with a diet supplemented with 0.2% (w/w) CPZ for six weeks and intraperitoneal injection of DAP (12.5 mg/kg/day, once daily) (Sigma, Aldrich) dissolved in normal saline (31-33) for the last two weeks of treatment with CPZ. The bodyweight of animals was measured every week.

***Behavioral tests***

The behavioral tests – pole test and rotarod performance test– were done on the last day of research to assess the locomotor coordination of all animals (training of mice was performed)(16). Both tests were done, scored, and evaluated blindly. For the pole test, each mouse was placed on the apex of the vertical pole (8 mm×55 cm), and the time to touch the floor was recorded. The cut-off time of the test was 1 min. 3 sequential trials were carried out every 5 min for each mouse.

The rotarod performance test was carried out with a rotarod device (Borj Sanat Azma, Tehran, Iran). The rotating rod had a 3 cm diameter and a height of 15 cm above the instrument floor. The speed was set at 25 rpm and 3 consecutive trials every 10 min were done. The animal was put on the rotating cylinder and was allowed to run. The latency to fall was recorded. The cut-off time of the test was 300 sec.

***Brain preparations***

At the end of the study, mice were anesthetized with ketamine/xylazine (50/10 mg/kg, IP). The brains of mice were carefully dissected, and half of them were fixed in 10% paraformaldehyde for hematoxylin and eosin** (**H & E) and myelin staining and immunohistochemistry (IHC) assays. The rest of the brains were placed in 1 ml of cold PBS (pH 7.4, 4 ^°^C) and homogenized by a homogenizer (Kunhewuhua, China) and then, centrifuged for 20 min (4 ^°^C, 12000 rpm). The supernatants were used for nitrite and SOD assays.

***Myelin staining***

Luxol fast blue staining (LFB) was used for staining axons on formalin-fixed, paraffin-embedded CNS tissue segments. The oligodendroglial myelin sheath on neurons, containing lipids, discolor from blue to green, and the neuronal cells discolor to purple. In this study, LFB was done to observe the region of demyelination in the corpus callosum based on the previous studies (34). After deep anesthesia in mice with ketamine and xylazine, the brain was removed. Then, a fixed corpus callosum was embedded in paraffin blocks. Then, 5 µm sections were cut by a microtome and incubated overnight at 56 ^°^C with LFB. Then, the slides were washed with 95% ethyl alcohol, differentiated in 0.05% LiCO_3_ solution for 30 sec and 70% ethyl alcohol for 30 sec, and rinsed in DW. Later, slides were dehydrated and fixed in graded ethyl alcohol and dimethyl benzene. The samples were checked by a light microscope (Olympus-ckx53), and the degree of demyelination was evaluated using the ImageJ software.

***Hematoxylin and eosin staining***

The fixed prefrontal cortex was embedded in a paraffin block. 5 µm sections were cut by a microtome and incubated with Harris’ hematoxylin solution (6 hr, 60 ^°^C). Then 10% acetic acid and 85% ethyl alcohol were used for tissue differentiation, and the tissues were washed with distilled water. After soaking the slides in lithium carbonate solution and washing with water, the staining was completed with eosin solution. The samples were checked by a light microscope, and vacuolization and chromatolysis of neurons were evaluated. 

***Immunohistochemistry staining***

To evaluate the presence of p-Nrf_2_ and p-IKB, the 5 µm thick prefrontal cortex pieces of mice were de-paraffinized with xylene. Then, the sections were hydrated in graded concentrations of ethanol and water. The sections were retrieved in citrate buffer (pH:7.4, 15 min, 90 ^º^C). After washing the slides with TBS plus 0.03% Triton X-100, blocking was performed with 1% BSA in TBS. Then, samples were blocked with a peroxidase blocking agent. Next, they were incubated at 4 ^°^C overnight with rabbit monoclonal p-Nrf_2_ (phospho S40) (1:100 dilution) antibody (Abcam, USA) and rabbit polyclonal p-IKB alpha (phosphor Ser32/S36) (1:100 dilution) antibody (Elabscience, USA). After washing with PBS and incubating with 3% H_2_O_2_ (15 min, RT), slides were incubated for one hour with goat biotinylated polyvalent secondary antibody (1:100 dilution) (Abcam, USA). The samples were stained with diaminobenzidine (DAB) (10 min) and water after washing with PBS. Counterstaining with hematoxylin (10 min, RT) and dehydrating with alcohol and xylene was performed. Finally, after applying the coverslip, the slides were observed and assayed with a light microscope. To quantify the results, ImageJ software was used (35). 

***Estimation of nitrite level and superoxide dismutase activity***

Nitrite levels of the whole brain supernatant were determined by the Griess reaction (36). The activity of SOD was measured following the procedure described by Misra and Fridovich (37).

***Data analysis***

Data are shown as mean±standard deviation (SD) using SPSS (21.0 for Windows, Chicago, IL, USA). The significance of the results was determined by one-way analysis of variance (ANOVA) followed by Tukey’s *post hoc* test to determine the differences between the groups involved.  *P*<0.05 was considered statistically significant.

## Results

***Bodyweight***

**Figure 1:**
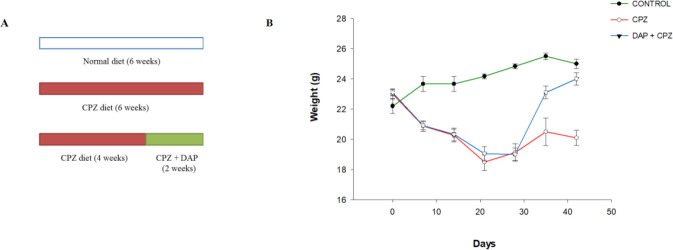
The body weight changes (n=7)

The bodyweight of animals was evaluated every week (Figure 1 A-B). After one week, animals from the CPZ group presented a lower body weight compared with the control group (*P*<0.05). Throughout the study up to 6 weeks, the bodyweight of the CPZ group remained significantly below that of the control group (*P*<0.001). DAP administration significantly reversed CPZ-induced weight loss in the last two weeks of the study compared with the CPZ group (*P*<0.001).

***Behavioral tests***

*Pole test*

**Figure 2:**
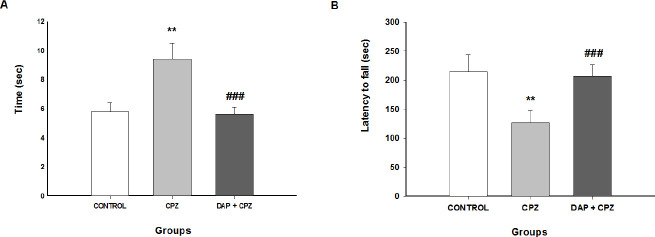
Behavioral tests (n=7)

The results of the pole test performed on the last day of the study are presented in Figure 2A. Feeding CPZ significantly increased the latency time to fall compared with the control diet (F (2, 69)=24.29, *P*<0.0001) though the latency was significantly shorter in the CPZ group treated with DAP than in the CPZ group (*P*<0.0001).

*Rotarod performance test*

The results from the rotarod performance test after 6 weeks are shown in Figure 2B. The time to fall was meaningfully reduced in the CPZ group compared with the control group (F (2, 60)=9.157, *P*=0.0014), indicating that the CPZ-treated mice had lower balance and coordination than mice receiving a normal diet. Additionally, treatment with DAP significantly increased the time to fall (*P*=0.0012), suggesting that DAP administration improved motor coordination. 

***Myelin staining (Luxol fast blue staining)***

**Figure 3:**
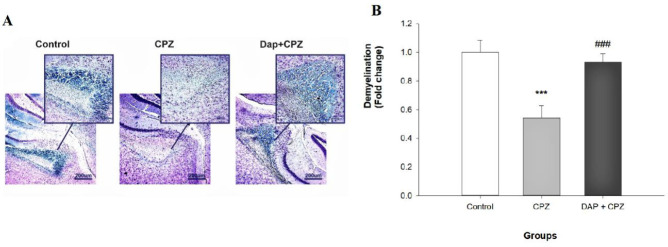
LFB staining of the corpus callosum (×100)

In this experiment, as shown in Figure 3 A-B, CPZ feeding resulted in massive demyelination in the corpus callosum compared with the control group (F (2, 12)=71.82, *P*<0.0001) evidenced by significant reduction of the intensity of blue color at the end of the study. DAP treatment showed a significant protective effect on myelin injury induced by CPZ and ameliorated the demyelination process observed in the CPZ group (*P*<0.0001). The intensity of the blue color is close to the color observed in control, indicating a good state of myelination. The integral optical densities of blue color in the three experimental groups are shown in Figure 3B.

***Hematoxylin and eosin staining***

**Figure 4:**
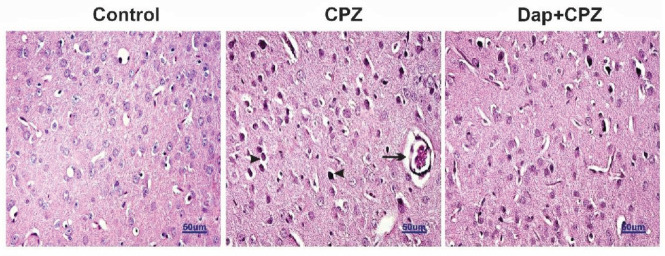
Photomicrograph of prefrontal cortex hematoxylin and eosin staining (×100). Dystrophy of neurons including chromatolysis and irregular Nissl granule distribution (arrows) and vacuolated neurons (arrowheads) in the CPZ group is shown, but neurons in the control and the DAP+CPZ group are nearly normal. (n=4)

CPZ: Cuprizone; DAP: Dapsone

To evaluate the oligodendrocyte and neuron structures, H & E staining of the prefrontal cortex was carried out. As shown in Figure 4, the CPZ group showed dystrophic alterations of neurons such as chromatolysis, abnormal Nissl granules, hyperchromatic nuclei, and vacuolated neurons. DAP administration reduced the pathological effects of CPZ in the cerebral cortex and showed proximate normal features.

***Immunohistochemistry staining of p-Nrf***_2_ ***and p-IKB***

**Figure 5:**
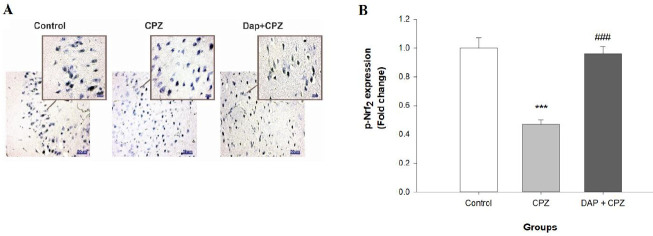
A: Photomicrograph of anti p-Nrf2 immunohistochemistry staining (IHC) of the prefrontal cortex (×100). B: Quantification of the IHC results. (n=4). ****P<0.0001 vs the control group, ^####^*P<*0.0001 vs the CPZ group

Immunohistochemical staining of p-Nrf_2_ and p-IKB was done to estimate the effect of CPZ and DAP treatment on these transcription factors. As shown in Figure 5 A-B, 6 weeks’ exposure to CPZ significantly reduced the levels of p-Nrf_2_ compared with the control group (F (2, 6)=182.8, *P*<0.0001), evidencing the existence of oxidative damage induced by CPZ. DAP administration significantly reduced the CPZ-induced alterations in the levels of p-Nrf_2_ (*P*<0.0001).

**Figure 6:**
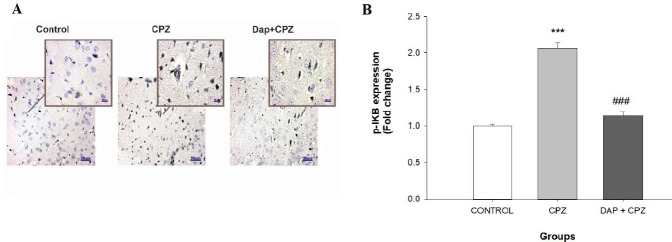
A: Photomicrograph of anti-p-IKB immunohistochemistry staining (IHC) of the prefrontal cortex (×100). B: Quantification of the IHC results. (n=4). ****P<0.0001 vs the control group, ^####^*P<*0.0001 vs the CPZ group

Another transcription factor that is influenced by CPZ is NF-kB, which increases the expression of pro-inflammatory cytokines (15). IKB forms a complex with NF-kB and inhibits its activity, but when IKB is phosphorylated (p-IKB), it dissociates from NF-kB, and NF-kB is activated (38). Figure 6 A-B shows that 6 weeks of CPZ feeding led to a significant elevation of p-IKB (an inactive form of IKB) (F (2, 6)=726.7, *P*<0.0001), which means that CPZ induces the activation of the NF-kB signaling pathway. DAP treatment significantly reduced the levels of p-IKB (*P*<0.0001).

***Nitrite level and superoxide dismutase activity***

Table 1 shows that CPZ significantly induces the nitrite levels of the brain compared with the control group (F (2, 9)=49.09, *P*=0.0097), and DAP administration significantly reversed the CPZ-induced changes in nitrite (*P*<0.0001). Oxidative stress was measured by evaluating the activity of the antioxidant enzyme SOD in brain tissue. As indicated in Table 1, CPZ administration during 6 weeks significantly reduced SOD activity (F (2, 9)=41.49, *P*=0.0002). Then, DAP treatment significantly reversed this reduced SOD activity (*P*<0.0001). These data indicate that CPZ exposure induced oxidative stress injury, whereas DAP ameliorated the CPZ toxicity. 

## Discussion

The main hallmark of MS is the demyelination of neuronal axons that occurs in the CNS (39). There are several *in vivo* models for studying myelin disorders, for example, experimental autoimmune encephalomyelitis (EAE) (40), virus-induced inflammatory demyelination (41), and CPZ induced demyelination (42, 43). The best described demyelinating model is the C57BL/6J mice fed 0.2% CPZ for 6 weeks, causing oligodendrocyte death and subsequent reversible demyelination (42). Despite types of research on CPZ-induced demyelination, the mechanism of its toxicity on oligodendrocytes is not exactly clear. It has been suggested that as CPZ is a copper chelator, it could inhibit Cu-dependent enzymes such as cytochrome oxidase and monoamine oxidase, altering energy metabolism and inducing apoptosis in oligodendrocytes (44, 45). Dap, an anti-microbial agent, is used for many inflammatory conditions such as acne vulgaris and dermatitis herpetiformis (46). DAP shows an anti-inflammatory effect via reducing pro-inflammatory cytokines, prostaglandins, and leukotrienes (24). In this study, CPZ was used as a model to evaluate the efficacy of DAP against the demyelination induced by CPZ in C57BL/6J mice through analyzing Nrf_2 _and NF-kB. Previous studies have demonstrated positive effects of DAP against renal ischemia-reperfusion damage, colitis, polycystic ovary syndrome (PCOS), bleomycin-induced idiopathic pulmonary fibrosis, and cardiotoxicity induced by doxorubicin via NF-kB targeting, leading to modulation of diverse oxidative stress and inflammatory mediators such as SOD, nitrite, MDA, and TNF-α (25, 28, 31, 47, 48). In another *in vivo* study on balb/c mice, DAP showed anti-inflammatory effects on ovalbumin-induced allergic rhinitis via reducing serum levels of IgE, IL-4, and IFNγ as well as decreased number of mucosal eosinophils and pathological changes in respiratory epithelial (49). It has been indicated that DAP has a protective effect on indomethacin-induced gastric ulcers in mice by targeting NF-kB, TNF-α, IL-1β, and decreasing oxidative stress biomarkers (50). In addition, DAP reduced memory impairment induced by scopolamine through modulating the NO pathway which was reversed by co-administration of NOS inhibitors in rats (51). 

Our data showed that the 6-week food supplemented with 0.2% (w / w) CPZ resulted in a deficit of locomotor coordination in the pole and rotarod performance tests. Many observations using the same model illustrated that dysfunctions in behavioral and motor tests are related to the demyelination of neurons (52, 53). To assay the effect of CPZ on demyelination of neurons, the corpus callosum, a myelin-rich area of the CNS, was analyzed. According to our results, CPZ intoxication leads to strong demyelination after 6 weeks. Conversely, treatment with DAP for the last 2 weeks of the study recovered the locomotor function. It improved the coordination and balance as well as improved remyelination of neurons in the corpus callosum of mice. In addition, the CPZ diet showed neurotoxic effects evidenced by chromatolysis, abnormal Nissl granules, hyperchromatic nuclei, and vacuolated neurons in the prefrontal cortex that were also ameliorated by DAP treatment. Previous studies have demonstrated that CPZ administration resulted in prefrontal cortex and hippocampus damage-inducing pyknosis, central chromatolysis, and cellular fragmentations (54, 55). The changes in the cellular morphology may be linked to elevation of oxidative stress mediators such as superoxide anion and nitrite, and that can damage neurons and apoptosis (56, 57). Evidence recommends that oxidative stress has a potential role in the pathophysiology of MS (58). CPZ leads to mitochondrial dysfunction (mitochondrial swelling, ROS generation, collapse of the membrane potential), and contributes to increased free radical production in oligodendrocytes, which can induce apoptosis (59-61).

In addition to the increase in nitrite production, CPZ reduced SOD activity in the brain. This reduction was reversed in the group treated with DAP for 2 weeks. It has been shown that activated microglia cause neurotoxic astrocytes and subsequently cause the death of nerve cells and oligodendrocytes (62). In addition, microglial activation is related to neurotoxicity via the release of pro-oxidant and pro-inflammatory agents such as ROS, NO, TNF-α, and IL-1β (63). One of the main inflammatory signaling pathways in oligodendrocytes is the NF-kB pathway which can be activated by CPZ intoxication (64, 65). Furthermore, analysis of the brains of patients with MS showed elevated expression of genes associated with NF-kB (66). NF-kB is a complex protein in the cytoplasm consisting of Rel and p50 subunits (67). In its inactive form, NF-KB is bound to the IKB in the cytosol. Due to phosphorylation and dissociation of p-IKB, NF-kB translocates to the nucleus and induces expression of pro-inflammatory mediators such as TNF-α, IL-1β, and iNOS (68, 69). In the present study, the CPZ diet increased the phosphorylation of IKB, leading to NF-kB activation and inflammation that contribute to neuronal and oligodendrocyte injury. It was established that one of the pathways favoring the demyelination process in the corpus callosum caused by CPZ is the NF-kB signaling pathway (70). DAP treatment during the last 2 weeks of the experimental procedure significantly reduced p-IKB levels suggesting reduced activation of NF-kB and inflammation. Previous studies have reported the inhibitory effects of DAP on NF-kB function and reduction of pro-inflammatory cytokines (21, 25). 

Nrf_2_, a transcription factor, has a cytoprotective role via increasing the expression of antioxidant genes, for instance, the detoxifying enzymes NQO-1 and HO-1 (71). Amira and colleagues have demonstrated that the CPZ diet caused a marked decline in the expression of Nrf_2_ and its downstream target genes in the brain (15). Besides, activation of NF-kB has an inhibitory effect on the expression of Nrf_2,_ contributing to oxidative stress and cell damage (72). The present results showed that CPZ exposure significantly decreased the active form of Nrf_2_ (p-Nrf_2_) associated with the critical role of this signaling in demyelination induced by CPZ. It has been evidenced that the Nrf_2_-deficient mouse exhibited a higher susceptibility toward CPZ toxicity and demyelination. DAP administration up-regulated the levels of p-Nrf_2_ and, consequently, could ameliorate oxidative stress and neuroinflammation. Accordingly, DAP administration could protect oligodendrocytes against oxidative stress injury by triggering the Nrf_2_ pathway. It has been evidenced that astrocytes and oligodendrocytes overexpressing Nrf2 are capable of saving neuronal cells from oxidative stress (73). Rios and his colleagues and Diaz-Ruiz *et al*. in separate studies have shown that DAP (12.5 mg/kg) reduced apoptosis in the spinal cord injury model in rats via decreasing the levels of caspase-8, 9, and 3 as well as acting as an antagonist of glutamate receptors, MPO activity, and improved motor function and coordination (74, 75). To clear whether DAP acts by inhibiting the death of oligodendrocytes and reducing inflammation or by actually contributing to remyelination, more cell culture studies may be useful.

Control: The control group receiving normal diet; CPZ: cuprizone group receiving diet supplemented with 0.2% (w/w) cuprizone for 6 weeks; DAP+CPZ: dapsone treated CPZ group receiving cuprizone diet for 6 weeks and the last 2 weeks dapsone administration intraperitoneally

CPZ: Cuprizone; DAP: Dapsone

A: Pole test, the graph indicates the latency time to touchdown; B: Rotarod test, the graph indicates the latency time to fall. ****P<0.0001 vs the control group, ^####^*P<*0.0001 vs the CPZ group

CPZ: Cuprizone; DAP: Dapsone

A: The control and DAP+CPZ group shows normal myelin in the corpus callosum (blue color) but demyelination in the CPZ group occurred (reduced blue intensity color). B: Quantification of the LFB results. (n=4) ****P<0.0001 vs the control group, ^####^*P<*0.0001 vs the CPZ group CPZ: Cuprizone; DAP: Dapsone; LFB:Luxol fast blue staining

CPZ: Cuprizone

CPZ: Cuprizone

**Table 1:** Brain levels of nitrite and superoxide dismutase

Examination	SOD activity (U/ml)	NITRITE (µM)
CONTROL	2.235 ± 0.007	6.440 ± 0.23
CPZ	2.180 ± 0.014^***^	7.60 ± 0.63^**^
DAP + CPZ	2.246 ± 0.004^####^	4.715 ± 0.21^####^

Control: control group, CPZ: CPZ group, DAP+CPZ: dapsone treated-CPZ group. **P<0.01, ***P<0.001 vs the control group; ^####^*P<*0.0001 vs the CPZ group

CPZ: Cuprizone; DAP: Dapsone

## Conclusion

The present study offers evidence for the neuroprotective effect of DAP against demyelination and locomotor dysfunction induced by CPZ. The protective effect of DAP may be correlated to its antioxidant and anti-inflammatory effects protecting oligodendrocytes from the oxidative stress induced by CPZ. The mechanism of action seems to be linked to the modulation of Nrf_2_ and NF-kB signaling pathways. Findings suggest that DAP may be a promising agent for remyelination, although clinical trials are necessary to assess the effectiveness of DAP in patients with MS.

## Authors’ Contributions

SS designed the research; AD, SS, and EK collected data; TN, AM-F, and LD analyzed data; AS and ES wrote the manuscript and created all the figures. All authors were involved in writing and revising the final manuscript. 

## Conflicts of Interest

The authors declare that they have no conflicts of interest.
